# Cadherin-11 serves as a novel receptor for *Fusobacterium nucleatum* adhesin FadA to exacerbate pulmonary inflammation

**DOI:** 10.1371/journal.ppat.1014158

**Published:** 2026-04-20

**Authors:** Kun Liu, Xinyun Xie, Yuchao Li, Shuwei Zhang, Wenli Zang, Qian Li, Yaping Pan

**Affiliations:** 1 Department of Periodontics, School and Hospital of Stomatology, China Medical University, Liaoning Provincial Key Laboratory of Oral Diseases, Shenyang, Liaoning Province, China; 2 Department of Oral Biology, School and Hospital of Stomatology, China Medical University, Liaoning Provincial Key Laboratory of Oral Diseases, Shenyang, Liaoning Province, China; University of Vermont, UNITED STATES OF AMERICA

## Abstract

*Fusobacterium nucleatum*, a periodontal pathogen, has been increasingly implicated in pulmonary diseases including chronic obstructive pulmonary disease (COPD). This study demonstrates that *F. nucleatum* adheres to and invades pulmonary epithelial cells in a dose-dependent manner, primarily mediated by its adhesin FadA. We identify cadherin-11 (CDH11), which is upregulated in COPD lungs and in pulmonary epithelial cells treated with *F. nucleatum* or FadAc protein, as the key host receptor for FadA. This FadA–CDH11 interaction not only mediates bacterial adhesion and invasion, but also activates the MAPK13/JUN pathway, leading to significant upregulation of pro-inflammatory cytokines including CSF3, TNF-α, CCL20, and TGF-β. Genetic knockdown of CDH11 abolishes MAPK13/JUN activation and cytokine induction but does not affect FadA-mediated p53 suppression, indicating a separate pathway for this oncogenic event. Our findings establish the FadA–CDH11–MAPK13/JUN axis as a central mechanism driving *F. nucleatum*-exacerbated pulmonary inflammation and tissue damage, highlighting its potential as a therapeutic target for mitigating COPD progression.

## Author summary

In this study, we investigated how *Fusobacterium nucleatum*, a bacterium commonly associated with periodontal disease, contributes to the worsening of pulmonary diseases such as chronic obstructive pulmonary disease (COPD). We discovered that a specific protein on the surface of this bacterium, called FadA, binds to a receptor protein in human pulmonary epithelial cells known as cadherin-11 (CDH11). This binding not only mediates bacterial adhesion and invasion, but also activates MAPK13/JUN inflammation pathways in pulmonary epithelial cells, leading to increased release of harmful molecules including CSF3, CCL20, TNF-α, and TGF-β, which can damage lung tissue and accelerate COPD progression. Importantly, we showed that blocking the interaction between FadA and CDH11 can reduce inflammation, suggesting a promising new therapeutic strategy for slowing down COPD in patients infected with *F. nucleatum*. Our work highlights the important connection between oral health and respiratory disease and offers a potential target for future therapies aimed at mitigating the impact of oral bacteria on lung health.

## Introduction

Chronic obstructive pulmonary disease (COPD) is an inflammatory lung disease that is characterized by emphysema and irreversible airflow restriction, symptoms that are likely result from chronic inflammation in the lungs [1]. Repeated exacerbations of COPD, which is frequently caused by bacterial or viral infection, accelerate the progression of emphysema and increase the risk of death. COPD has become the fourth leading cause of mortality [2], with approximately 3 million people dying from COPD each year worldwide [3]. Therefore, effective prevention of COPD exacerbations has important implications for clinical management and public health.

Periodontitis is a major oral disease and a prevalent global health issue, primarily driven by dysbiotic microbial biofilms containing pathogens such as *Porphyromonas gingivalis* and *Fusobacterium nucleatum* [4–6]. It leads to progressive destruction of periodontal tissues and has recently been recognized as an independent risk factor for COPD exacerbation [7,8]. This association is attributed to shared inflammation and the translocation of oral bacteria, which may reach the lungs through aspiration, thereby worsening pulmonary inflammation and contributing to COPD progression [9–14]. *F. nucleatum*, a gram-negative obligate anaerobe prevalent in dental plaque biofilms of patients with periodontitis [15,16], is detected in sputum and bronchoalveolar lavage fluid (BALF) of COPD patients, where its presence correlates with disease severity [17–19]. In elastase-induced emphysematous mice, intratracheal administration of *F. nucleatum* exacerbated inflammatory responses, increased the production of alveolar wall destruction factors MMP-12 and perforin, promoted mucin recruitment, and accelerated emphysema progression [20]. Similarly, Li et al. found that *F. nucleatum* translocated from oral cavity to lungs, where it induced bullae formation, pronounced inflammatory infiltration, and upregulated IL-1β, IL-6, TNF-α, IFN-γ, MMP-8, and neutrophil elastase in lung tissues, driving COPD-like lung changes in mice [10]. Our previous study further demonstrated that *F. nucleatum* could adhere to and invade pulmonary epithelial cells, and aggravate the infection and inflammatory damage caused by the respiratory pathogen *Pseudomonas aeruginosa* [21]. Nevertheless, the molecular mechanisms by which *F. nucleatum* adheres to and invades pulmonary epithelial cells, as well as how it triggers pulmonary inflammation, remain unclear.

FadA is a unique adhesin exclusively expressed on the surface of oral *Fusobacterium* species [22], and is critical for bacterial adhesion and colonization of host cells [23–27]. It exists in two forms: non-secreted pre-FadA, anchored to the bacterial inner membrane, and secreted mature FadA (mFadA), exposed on the bacterial surface. These assemble at a specific ratio to form the FadA complex (FadAc), which adopts a short filamentous or knot structure that mediates *F. nucleatum* adhesion to and invasion of host cells [23,24,28]. Pretreatment of host cells with FadAc competitively inhibits *F. nucleatum* adhesion and invasion in oral keratinocytes (OKF6/Tert), Chinese hamster ovary (CHO) cells, and colorectal cancer (CRC) cells [23,24,28], while *fadA* deletion reduces bacterial binding to CHO and OKF6/Tert cells by 70–80% [22,23] and prevents bacterial translocation across the placental barrier [25]. Based on these findings, we aim to investigate whether FadA also plays a critical role in *F. nucleatum* adhesion to and invasion of pulmonary epithelial cells.

Cadherins are a large family of calcium-dependent transmembrane glycoproteins that mediate homophilic cell-cell adhesion, and play essential roles in tissue morphogenesis and signal transduction [29]. The main branch includes classical (type I) cadherins (such as CDH1, CDH2, CDH3, CDH4, CDH15, and CDH16), and atypical (type II) cadherins (including CDH5, CDH6, CDH7, CDH8, CDH9, CDH10, CDH11, CDH12, CDH17, CDH18, CDH19, CDH20, CDH22 and CDH24) [30]. FadA has been shown to bind the 415–534 region of vascular endothelial cadherin (CDH5/VE-cadherin), facilitating *F. nucleatum* adhesion to and invasion of vascular endothelial cells [31]. This interaction disrupts endothelial integrity by displacing CDH5 from cellular junctions, thereby increasing permeability and facilitating bacterial dissemination across the placental barrier [31]. Similarly, Rubinstein et al. found that FadA targets the extracellular domain 5 (EC5) of epithelial cadherin (CDH1/ E-cadherin) in CRC cells, enabling *F. nucleatum* to adhere to and invade CRC tissues [27,32]. These findings suggest that during pulmonary infection, FadA may employ an analogous mechanism—targeting cadherin receptors on pulmonary epithelial cells to compromise the host barrier and promote invasion.

Persistent airway inflammation triggered by bacterial infection is a critical driver of COPD exacerbation. As a major member of the MAPK family, p38 mitogen-activated protein kinase (p38 MAPK) is primarily involved in cellular stress responses, particularly inflammation and oxidative stress. Increased phosphorylation of p38 has been detected in the sputum of COPD patients and is correlated with worsening airway inflammation and declining lung function [33]. Upon activation, p38 MAPK phosphorylates downstream kinases such as MAPK-activated protein kinase 2/3 (MAPKAPK2/3), ultimately modulating transcription factors, cytoskeletal proteins, translation-related components, and other enzymes. Our previous studies revealed that *F. nucleatum* and *P. aeruginosa* frequently coexist in the airways during acute exacerbations of COPD. Interestingly, while *F. nucleatum* attenuates *P. aeruginosa*-induced cytotoxicity, it synergistically enhances IL-6 production in pulmonary epithelial cells [21]. Animal models further underscore the pro-inflammatory role of *F. nucleatum*: intratracheal inoculation with *F. nucleatum* increased macrophage and neutrophil infiltration in BALF and upregulated TNF, IL-6, and CXCL1 in lung tissues of COPD mice [20]. Likewise, oral infection with *F. nucleatum* promoted inflammatory infiltration around bronchi and alveoli, accompanied by elevated expression of IL-1β, IL-6, TNF-α, MMP-8, and neutrophil elastase [10]. Therefore, this study aims to investigate whether *F. nucleatum*, through FadA-mediated binding to host cadherins, exacerbates pulmonary epithelial inflammation via activation of the MAPK13 cascade. Here, we identify CDH11 as the previously unknown host receptor for FadA to mediate *F. nucleatum* adhesion and invasion into pulmonary epithelial cells. Furthermore, we demonstrate that the FadA–CDH11 interaction activates the MAPK13/JUN signaling pathway, driving a pro-inflammatory cytokine response that may contribute to COPD pathogenesis.

## Results

### FadA mediates *F. nucleatum* adhesion and invasion of pulmonary epithelial cells

**Fig 1:**
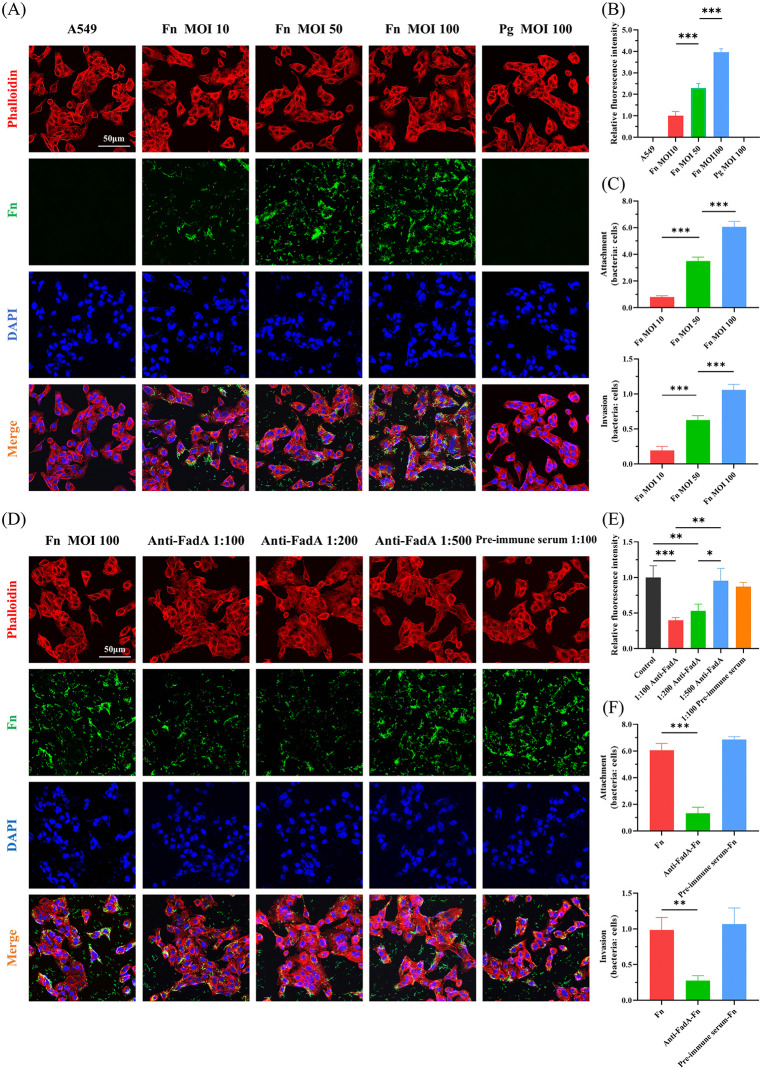
FadA mediates *F. nucleatum* adhesion and invasion of pulmonary epithelial cells. (A) Representative confocal microscopy images of *F. nucleatum* adhesion and invasion in pulmonary epithelial cells. A549 cells were infected with *F. nucleatum* at different MOI (10, 50, 100) or *P. gingivalis* at MOI 100. Green fluorescence indicates *F. nucleatum* labeled with specific antibody, blue fluorescence represents DAPI-stained nuclei, and red fluorescence shows actin cytoskeleton stained with phalloidin. Scale bar: 50 μm. (B) Quantification analysis of fluorescence intensity from (A). (C) Colony formation analysis of the adhesion and invasion of *F. nucleatum* in pulmonary epithelial cells. A549 cells were infected with *F. nucleatum* at different MOI (10, 50, 100). (D) Representative confocal microscopy images evaluating the effect of FadA antibody blockade on the adhesion and invasion of *F. nucleatum* to pulmonary epithelial cells. Prior to infecting A549 cells, *F. nucleatum* was treated with different dilutions of FadA antibody (anti-FadA; 1:100, 1:200, 1:500), using pre-immune serum (1:100) as a control. Actin cytoskeleton is shown in red, *F. nucleatum* in green, and nuclei in blue. Scale bar: 50 μm. (E) Quantification analysis of fluorescence intensity from (D). (F) Colony formation analysis of the effect of FadA antibody blockade on the adhesion and invasion of *F. nucleatum* to pulmonary epithelial cells. *F. nucleatum* was pre-treated with anti-FadA (1:100) or pre-immune serum (1:100). Data shown in (B, C, E and F) are presented as mean ± SD (n = 3 independent experiments). *P < 0.05, **P < 0.01, ***P < 0.001. Fn, *F. nucleatum*; Pg, *P. gingivalis*.

To investigate the role of FadA in host-pathogen interaction, we first assessed the dynamics of *F. nucleatum* infection. The immunofluorescence results showed that the *F. nucleatum* polyclonal antibody specifically labeled *F. nucleatum* (green fluorescence) infecting pulmonary epithelial cells, while no significant fluorescent signal was detected in *P. gingivalis*-infected pulmonary epithelial cells, indicating that the *F. nucleatum* polyclonal antibody exhibits excellent species specificity (Fig 1A). Second, as the MOI value of *F. nucleatum* increased, the green fluorescence on the surface and inside the pulmonary epithelial cells significantly intensified (Fig 1A and 1B), and the adhesion and invasion rates of *F. nucleatum* into the pulmonary epithelial cells also significantly increased (Figs 1C and S1A). As shown in S1B Fig, no bacterial colonies were formed from the cell culture supernatant after antibiotic treatment, demonstrating that the combination of gentamicin and metronidazole effectively killed extracellular *F. nucleatum*. Subsequently, *F. nucleatum* was pretreated with different concentrations of FadA antibody (1:100, 1:200, 1:500), with pre-immune serum (1:100) serving as negative control. Figs 1D-1F and S1C demonstrated that FadA antibody (1:100 and 1:200) significantly inhibited the adhesion and invasion of *F. nucleatum* into pulmonary epithelial cells, whereas pre-immune serum (1:100) had no effect on the adhesion and invasion rates of *F. nucleatum*. In addition, we used FadA antibody to label the FadA protein (green fluorescence) that interacts with pulmonary epithelial cells, and no significant fluorescence signal was detected in *P. gingivalis*-infected pulmonary epithelial cells, confirming excellent species specificity of FadA antibody (S2A Fig). The level of FadA protein interacted with pulmonary epithelial cells increased with higher bacterial MOI (S2A Fig), and *F. nucleatum* expressing FadA (green fluorescence in S2B and S2C Fig) could invade pulmonary epithelial cells (S2B and S2C Fig). These results indicate that FadA is a critical virulence factor mediating the adhesion and invasion of *F. nucleatum* into pulmonary epithelial cells.

### FadA-CDH11 binding initiates *F. nucleatum* adhesion and invasion into pulmonary epithelial cells

**Fig 2:**
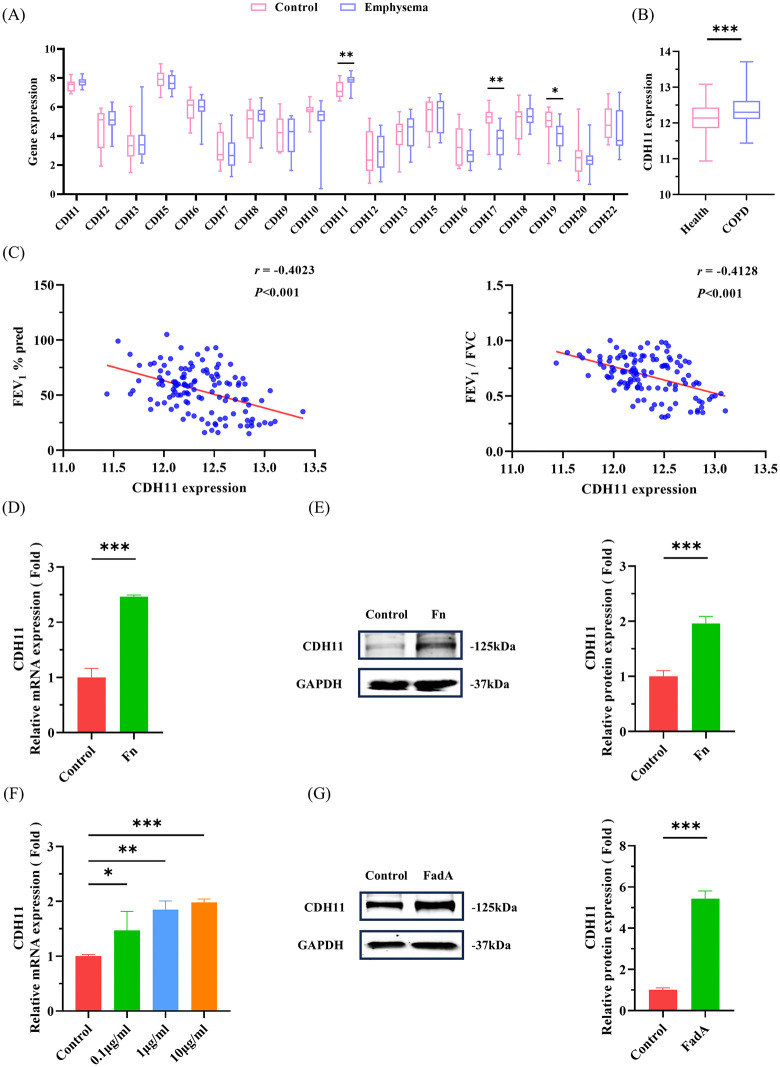
Cadherin expression profiles in COPD lungs and pulmonary epithelial cells treated with *F. nucleatum* and FadAc protein. (A) Gene expression of cadherins in lungs of COPD patients with severe emphysema (GSE1650). (B) Gene expression validation of CDH11 in lungs of COPD patients (GSE47460). (C) Correlation scatter plots of CDH11 expression with FEV_1_% pred and FEV_1_/FVC in lungs of COPD patients (GSE47460). The *r* and *P* values were calculated by Spearman correlation analysis. (D, E) QRT-PCR and western blot analysis of the effect of *F. nucleatum* on CDH11 mRNA **(D)** and protein **(E)** expression in pulmonary epithelial cells. A549 cells were infected with *F. nucleatum* (MOI 100). (F, G) QRT-PCR and western blot analysis of the effect of FadAc protein on CDH11 mRNA **(F)** and protein **(G)** expression in pulmonary epithelial cells. A549 cells were treated with the indicated concentrations of FadAc protein (0.1 μg/mL, 1 μg/mL and 10 μg/mL in **F**, and 10 μg/mL in G). Data shown in (D-G) are represented as mean ± SD (n = 3). *P < 0.05, **P < 0.01, ***P < 0.001.

**Fig 3:**
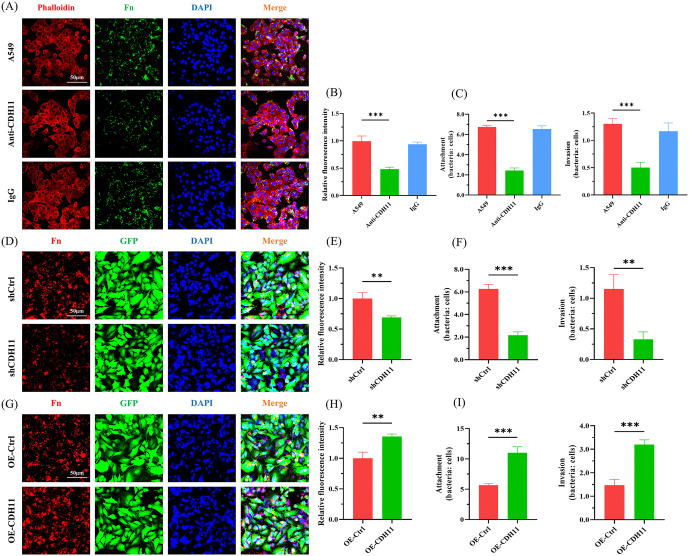
The effect of CDH11 on the adhesion and invasion of *F. nucleatum* in pulmonary epithelial cells. (A) Confocal microscopy analysis of *F. nucleatum* adhesion and invasion following CDH11 antibody blockade. A549 cells were pretreated with CDH11 antibody (10 μg/mL) or IgG isotype control (10 μg/mL), then infected with *F. nucleatum* (MOI 100). Red: actin cytoskeleton; green: Fn; blue: cell nucleus. Scale bar: 50 μm. (D, G) Confocal microscopy analysis of *F. nucleatum* adhesion and invasion in CDH11-knockdown or overexpressing cells. A549 cells with CDH11 knockdown (shCDH11) or CDH11 overexpressing (OE-CDH11) were infected with *F. nucleatum* (MOI 100). Red: Fn; green: A549 cells; blue: cell nucleus. Scale bar: 50 μm. (B, E, H) Quantification analysis of fluorescence intensity from (A), (D), and (G), respectively. (C, F, I) Colony formation analysis of the effect of CDH11 on the adhesion and invasion of *F. nucleatum* to pulmonary epithelial cells. In (C), A549 cells were pretreated with CDH11 antibody (10 μg/mL) or IgG isotype control (10 μg/mL), and infected with *F. nucleatum* (MOI 100). In (F) and (I), shCDH11 or OE-CDH11 cells were infected with *F. nucleatum* (MOI 100). Data shown in (B, C, E, F, H and I) are represented as mean ± SD (n = 3). **P < 0.01, ***P < 0.001. Fn, *F. nucleatum*.

To identify potential cadherin receptors mediating the adhesion and invasion of *F. nucleatum* into pulmonary epithelial cells, analysis of GSE1650 dataset demonstrated that CDH11 expression was significantly elevated, while CDH17 and CDH19 expression were markedly reduced in the lungs of COPD patients with severe emphysema (Fig 2A). This upregulation of CDH11 was validated in the independent GSE47460 cohort, which also showed significantly higher CDH11 levels in COPD lungs compared to controls (Fig 2B). To assess the clinical relevance of this finding, we examined the correlation between CDH11 expression and key pulmonary function parameters, namely the forced expiratory volume in one second percentage of predicted value (FEV_1_% pred) and the ratio of FEV_1_ to forced vital capacity (FEV_1_/FVC). In COPD patients, CDH11 expression exhibited a significant negative correlation with both FEV_1_% pred and FEV_1_/FVC (Fig 2C). Additionally, both *F. nucleatum* infection and FadAc protein stimulation significantly increased the expression of CDH11 mRNA and protein in pulmonary epithelial cells (Fig 2D–2G). These findings prompted us to further explore whether CDH11 serves as a critical mediator of *F. nucleatum* adhesion and invasion in pulmonary epithelial cells. To address this question, we established stable CDH11-knockdown (shCDH11) and CDH11-overexpressing (OE-CDH11) pulmonary epithelial cell lines through lentiviral transfection (S3A Fig). QRT-PCR and western bolt confirmed that shCDH11 reduced CDH11 mRNA and protein levels by 81.4% ± 2.1% (shCDH11–1), 94.3% ± 2.6% (shCDH11–2) and 10.6% ± 6.5% (shCDH11–3), and 51.3% ± 3.4% (shCDH11–1), 48.7% ± 4.2% (shCDH11–2) and 8.0% ± 6.1% (shCDH11–3), respectively (S3B and S3C Fig), while OE-CDH11 increased CDH11 mRNA and protein expression 2.3 ± 0.2-fold and 2.1 ± 0.2-fold (S3D and S3E Fig). Both immunofluorescence and colony counting assays demonstrated that CDH11 antibody blockade and CDH11 knockdown inhibited the adhesion and invasion of *F. nucleatum* into pulmonary epithelial cells, while CDH11 overexpression enhanced the adhesion and invasion of *F. nucleatum* into pulmonary epithelial cells (Figs 3 and S4). Notably, neither knockdown nor overexpression of CDH11 altered the expression of other critical adhesion molecules including occludin, CDH1, CDH5, and integrin α2 (ITGA2) in pulmonary epithelial cells (S5 Fig), which indicates a distinct and specific contribution of CDH11 to host-bacterial interaction.

**Fig 4:**
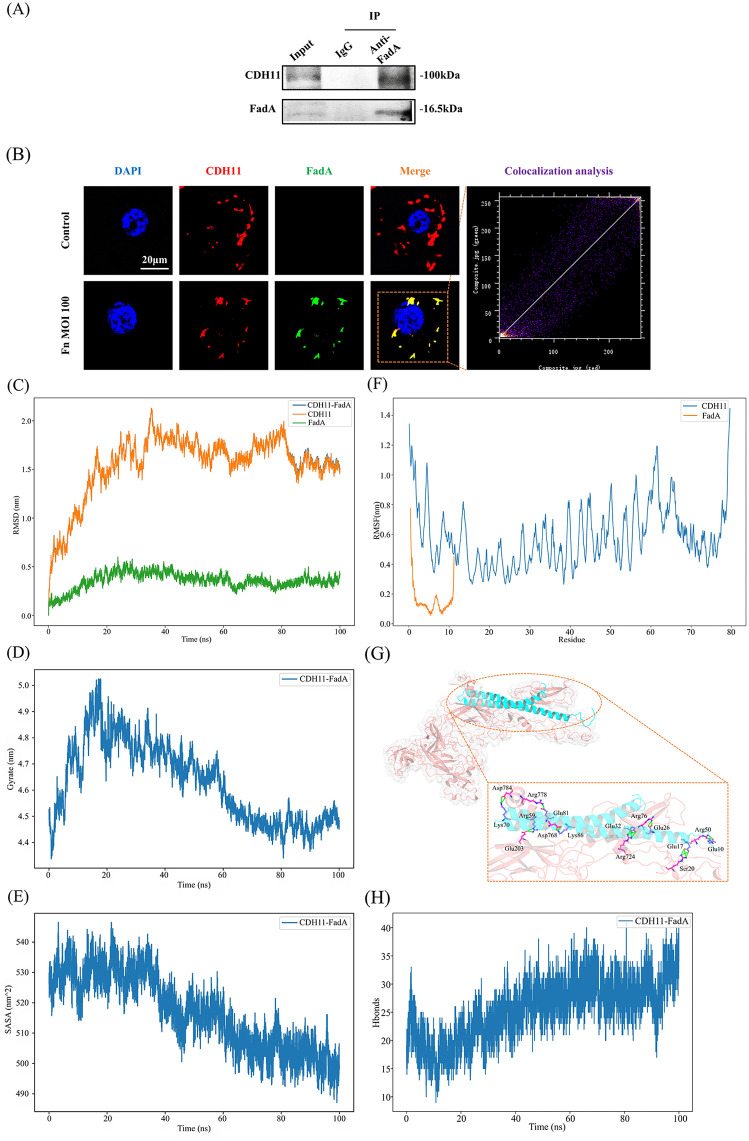
FadA interacts with CDH11. (A) Co-IP analysis of the interaction between CDH11 and FadA. (B) Fluorescence colocalization analysis of CDH11 and FadA in A549 cells. Representative confocal microscopy images showing the subcellular distribution of FadA (green) and CDH11 (red). Nuclei are stained with DAPI (blue). Yellow regions in merged panels indicate colocalization. Quantitative analysis of colocalization was measured by Pearson’s correlation coefficient (PCC). Scale bar: 20 μm. (C–H) Stability analysis of the FadA–CDH11 complex from all-atom MD simulations: (C) RMSD analysis of the protein backbones for CDH11 (orange), FadA (green), and the complex (blue) during the MD simulation. (D) Rg analysis of the FadA-CDH11 complex in MD simulations. (E) SASA analysis of the complex over the simulation time. (F) RMSF analysis of CDH11 (blue) and FadA (orange) for each residue. (G) 3D interaction plot of the FadA–CDH11 complex. Amino acid residues of FadA and CDH11 are colored in blue and pink, respectively. Hydrogen bonds and salt bridges are colored in green. (H) Number of intermolecular hydrogen bonds between FadA and CDH11 over the MD simulation trajectory.

On the other hand, as shown in the amino acid sequence alignment (S6 Fig), CDH11 exhibits significant sequence homology with the two known FadA receptors, CDH1 and CDH5. CDH11 shares 42.3% identity and 61.7% similarity with CDH1 (the FadA receptor in CRC cells), and 38.9% identity and 57.2% similarity with CDH5 (the FadA receptor in vascular endothelial cells). This degree of conservation among these classical cadherins suggests a common structural basis that may underlie FadA recognition across different host cell types. Co‐immunoprecipitation demonstrated a specific interaction between FadA and CDH11 in pulmonary epithelial cells (Fig 4A). Immunofluorescence co-localization assays further revealed significant overlap between FadA and CDH11 on the plasma membrane of pulmonary epithelial cells (R = 0.97 by Pearson’s correlation; Fig 4B). Furthermore, molecular docking (GRAMM) identified a stable FadA-CDH11 binding pose, which was further validated by molecular dynamics (MD) simulation to assess dynamic stability (Fig 4C–4H). Given CDH11’s multi-domain architecture with long, flexible loops, we first assessed the global conformational stability of the system: the root-mean-square deviation (RMSD) of the CDH11-FadA complex (Fig 4C) gradually converged to 1.5–2.0 nm after 80 ns (primarily driven by flexible linker motions), while CDH11 alone exhibited higher RMSD (peaking at 2.0 nm), and FadA remained relatively stable (RMSD <0.5 nm). To rule out non-physical unfolding, the radius of gyration (Rg) decreased from 5.0 nm to 4.5 nm by 60 ns (Fig 4D), indicating domain reorganization from an extended to a thermodynamically favorable compact state. Concurrently, the solvent-accessible surface area (SASA) converged smoothly (Fig 4E), confirming thermodynamic equilibration. For local flexibility and binding integrity, residue-level root mean square fluctuation (RMSF) analysis (Fig 4F) revealed that CDH11’s high fluctuations (>0.8 nm) were restricted to its surface long loops, whereas its folded core remained rigid. In contrast, FadA (an α-helical protein) exhibited minimal fluctuation (<0.2 nm) with stable RMSD (0.3–0.5 nm), indicating FadA’s persistent anchoring in the binding pocket despite CDH11’s large-scale conformational changes. The key interaction network (Fig 4G) extracted from the equilibrated MD phase (80–100 ns) showed dense hydrogen bonds and salt bridges (e.g., Arg76 of CDH11 and Glu26 of FadA, Glu203 of CDH11 and Arg59 of FadA). Trajectory statistics (Fig 4H) further confirmed persistent intermolecular hydrogen bonds (fluctuating 15–30 with high occupancy). Finally, binding free energy calculations using the Molecular Mechanics/Generalized Born Surface Area (MM/GBSA) method yielded a net ΔTotal of -195.47 ± 0.96 kcal/mol (Table 1), demonstrating that the FadA-CDH11 interaction is thermodynamically feasible. These results suggest that FadA binds stably and specifically to CDH11, initiating *F. nucleatum* adhesion and invasion into pulmonary epithelial cells via a direct, energetically favorable interaction.

**Table 1:** Molecular docking results of FadA with CDH11.

Contribution components	CDH11-FadA (kcal/mol)
Δ_VDWAALS_	-297.73 ± 0.96
ΔE_elec_	1168.07 ± 3.93
ΔE_GB_	-1020.65 ± 3.61
ΔE_surf_	-45.15 ± 0.10
ΔG_gas_	870.33 ± 3.81
ΔG_solvation_	-1065.80 ± 3.61
ΔTotal	-195.47 ± 0.96

Δ_VDWAALS_, van der Waals energy; ΔE_elec_, electrostatic energy; ΔE_GB_, generalized born solvation energy; ΔE_surf_, surface energy; ΔGgas, gas-phase free energy; ΔG_solvation_, solvation free energy, ΔTotal, the total binding free energy.

### *F. nucleatum* activates MAPK13/JUN inflammatory pathway in pulmonary epithelial cells via FadA-CDH11 interaction

**Fig 5:**
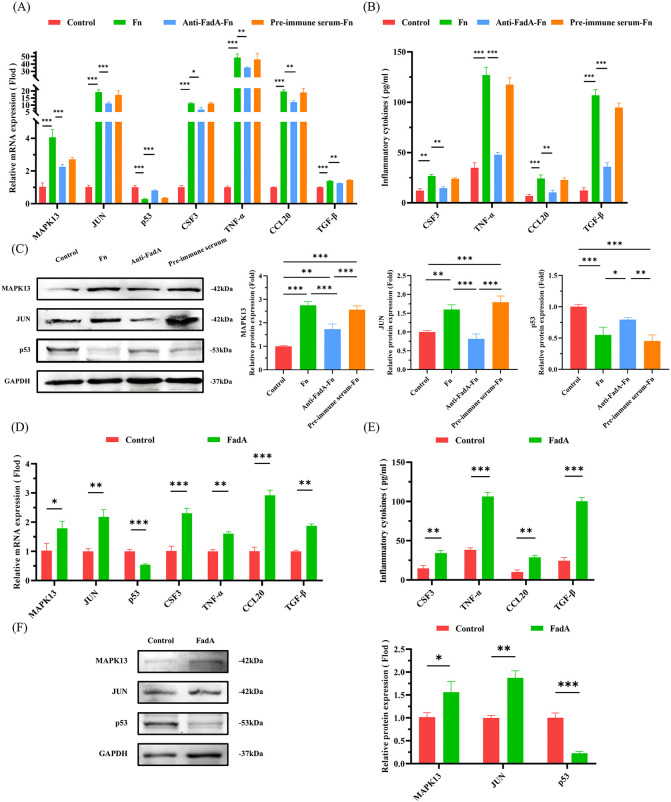
*F. nucleatum* activates MAPK13/JUN inflammatory pathway via FadA in pulmonary epithelial cells. **(A, D)** QRT-PCR analysis of the effects of *F. nucleatum* (A) or FadA (D) on the mRNA expressions of MAPK13, JUN, CSF3, TNF-α, CCL20, and TGF-β in pulmonary epithelial cell. **(B, E)** ELISA analysis of the effects of *F. nucleatum* (B) or FadA (E) on the protein expression of CSF3, TNF-α, CCL20, and TGF-β. **(C, F)** Western blot analysis of the effects of *F. nucleatum* (C) or FadA (F) on the protein expression of MAPK13, JUN, and p53. A549 cells were infected with *F. nucleatum* (MOI 100, A-C), anti-FadA pre-treated *F. nucleatum* (MOI 100, anti-FadA 1:100, A-C), and FadAc protein (10 μg/mL, D-F). Data in Fig 5 are represented as mean ± SD, n = 3 independent samples. *P < 0.05, **P < 0.01, ***P < 0.001. Fn, *F. nucleatum*.

Having established the FadA-CDH11 interaction, we sought to determine whether FadA is responsible for activating downstream pro-inflammatory signaling pathways in pulmonary epithelial cells. Compared to the control group, *F. nucleatum* infection promoted the mRNA and protein expression of MAPK13, JUN, CSF3, TNF-α, CCL20, and TGF-β, while suppressing the mRNA and protein expression of p53 (Fig 5A–5C). Antibody blockade of FadA reversed the promotive effects of *F. nucleatum* on the expression of MAPK13, JUN, CSF3, TNF-α, CCL20, and TGF-β, as well as its inhibitory effect on p53 expression (Fig 5A–5C). In contrast, pretreatment of *F. nucleatum* with pre-immune serum did not produce such reversal effects (Fig 5A–5C). Moreover, treatment of pulmonary epithelial cells with FadAc protein (10 μg/mL) also promoted the mRNA and protein expression of MAPK13, JUN, CSF3, TNF-α, CCL20, and TGF-β, and suppressed the mRNA and protein expression of p53 (Fig 5D-5F). These results indicate that FadA adhesin is the primary virulence factor of *F. nucleatum* in driving MAPK13/JUN pro-inflammatory signaling while inhibiting p53.

**Fig 6:**
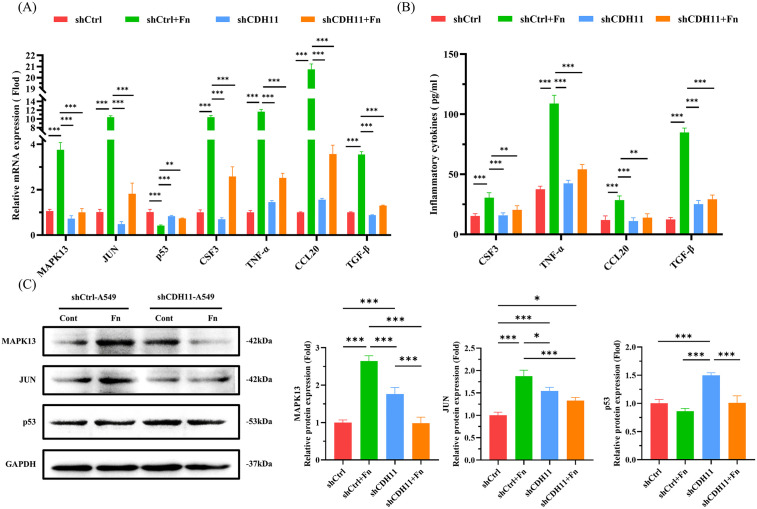
The effect of CDH11 on the MAPK/JUN inflammatory pathway in pulmonary epithelial cells. (A) QRT-PCR analysis of the effects of CDH11 on the mRNA expressions of MAPK13, JUN, CSF3, TNF-α, CCL20, and TGF-β in pulmonary epithelial cell. (B) ELISA analysis of the effects of CDH11 on the protein expression of CSF3, TNF-α, CCL20, and TGF-β. (C) Western blot analysis of the effects of CDH11 on the protein expression of MAPK13, JUN, and p53. A549 cells with CDH11 knockdown (shCDH11), along with the control (shCtrl), were infected with *F. nucleatum* (MOI = 100). Data in Fig 6 are represented as mean ± SD, n = 3 independent samples. *P < 0.05, **P < 0.01, ***P < 0.001. Fn, *F. nucleatum*.

On the other hand, knockdown of CDH11 markedly attenuated the promoting effects of *F. nucleatum* on the expression of MAPK13, JUN, CSF3, TNF-α, CCL20, and TGF-β, as evidenced by the significant difference from the virus vector control group (shCtrl, Fig 6). In contrast, the suppression of p53 by *F. nucleatum* remained unaffected in the CDH11 knockdown group compared to the shCtrl group (Fig 6). This intriguing result suggests that CDH11 is a critical receptor specifically mediating the MAPK13/JUN pro-inflammatory signaling pathway activated by FadA adhesin of *F. nucleatum*, but it is not involved in the pathway leading to p53 suppression.

## Discussion

*F. nucleatum*, a gram-negative obligate anaerobe, can be classified into several subspecies, including *F. nucleatum* subsp*. nucleatum*, *F. nucleatum* subsp*. polymorphum*, *F. nucleatum* subsp*. vincentii*, *F. nucleatum* subsp. *fusiforme*, and *F. nucleatum* subsp*. animalis* [34]. However, clinical metagenomic studies of respiratory infections typically identify *F. nucleatum* at the species level, leaving the relative contribution of different subspecies to respiratory diseases an open question [12–14,35]. *F. nucleatum* subsp. *nucleatum* ATCC 25586 (*F. nucleatum* ATCC 25586) has been established as the most primary and extensively studied model in the context of oral-pulmonary interactions. Our previous study demonstrated a 60.8% detection rate for *F. nucleatum* in the airways of patients with acute COPD exacerbation, with its abundance negatively correlating with pulmonary function [17]. *In vitro* and *in vivo* studies have demonstrated that *F. nucleatum* ATCC 25586 can adhere to and invade respiratory epithelial cells, provoke inflammatory responses, and induce COPD-like lung changes in mice [10,20,21,36,37]. However, the specific bacterial virulence factors and host effector proteins critically involved in this process remain unidentified. Therefore, this study continues to focus on the *F. nucleatum* ATCC 25586 to elucidate these key molecular determinants.

The respiratory mucosal epithelium constitutes the primary natural barrier of the host defense against microbial infections. Bacterial adhesion to and invasion of host cells are essential for their virulence and pathogenicity. Our previous studies demonstrated that *F. nucleatum* can adhere to and invade pulmonary epithelial cells, with efficiency increasing in a dose-dependent manner [21]. FadA, an adhesin exclusively expressed on the surface of oral *Fusobacterium* species, is critically involved in bacterial adhesion and invasion of host cells [22]. FadA mediates *F. nucleatum* adhesion to and invasion of vascular endothelial cells and CRC cells. Genetic deletion of *fadA* reduces bacterial binding to CHO and OKF6/Tert cells by 70–80%, while complementation with *fadA* restores bacterial binding to host-cell [22,23,25]. In this study, we confirmed that *F. nucleatum* adhered to and invaded pulmonary epithelial cells, with efficiency escalating as MOI increases. Meanwhile, FadA could enter pulmonary epithelial cells, and its intracellular amount rose proportionally with MOI. Pretreatment of *F. nucleatum* with FadA antibodies significantly reduced its adhesion and invasion. These results suggest that FadA mediates the adhesion and invasion of *F. nucleatum* to pulmonary epithelial cells, and the residual adhesion and invasion post-anti-FadA treatment may arise from incomplete FadA blockade or redundant adhesins such as Fap2 and FomA [38]. A key limitation of this work is the unsuccessful construction of a FadA mutant and its complemented strain. The primary challenge stemmed from the profound genetic intractability of the *F. nucleatum* ATCC 25586, which is mediated by its robust restriction-modification (R-M) systems. Although we implemented the host mimicry strategy described by Umaña et al. [39], which utilizes native DNA methylation to protect plasmid DNA from R-M system cleavage, our efforts to construct a stable FadA mutant were unfortunately unsuccessful.

Cadherins are a superfamily of calcium-dependent transmembrane cell-surface proteins that mediate homophilic cell-to-cell adhesion. The present study reveals a distinct cadherin expression profile in the lungs of COPD patients with severe emphysema, characterized by significantly elevated CDH11 expression concomitant with marked reduction of CDH17 and CDH19 (GSE1650). This cadherin switching pattern is likely to contribute to the loss of epithelial integrity characteristic of emphysema. Previous studies have established that CDH11 promotes fibroblast activation and epithelial-mesenchymal transition (EMT) in pulmonary fibrosis [40–42]. Our findings also point out that both *F. nucleatum* infection and purified FadAc protein stimulation significantly increased CDH11 expression in pulmonary epithelial cells. These results suggest that *F. nucleatum,* particularly its adhesin FadA, may contribute to alveolar destruction by inducing EMT-like changes in pulmonary epithelium via CDH11 upregulation, which warrants further investigation.

The mFadA monomer consists of two antiparallel α-helices connected by an 8-amino acid loop. Intramolecular and intermolecular leucine zipper motifs are present within and between mFadA molecules [24]. Mutations introduced in the loop region (F68A, Y69A, K70A) or at key leucine zipper residues (Leu14 and Leu76) were shown to impair the ability of FadA to bind to various host cells, including CHO cells, OKF6/Tert cells and human umbilical vein endothelial cells [24,28]. As a member of the cadherin superfamily alongside the known FadA receptors CDH1 and CDH5, CDH11 shares the conserved domain architecture of five extracellular cadherin repeats, a transmembrane region, and a cytoplasmic tail. The key distinction lies in their subtype: CDH1 is a type I cadherin containing one conserved extracellular tryptophan, whereas CDH5 and CDH11 are type II cadherins containing two. Notably, pretreatment of pulmonary epithelial cells with a CDH11-specific antibody significantly attenuated *F. nucleatum* adhesion and invasion, and genetic modulation of CDH11 expression (knockdown or overexpression) specifically altered the adhesion/invasion capacity of *F. nucleatum* without affecting the expressions of other major adhesion molecules such as occludin [43], CDH1 [44], CDH5 [45], and ITGA2 [46]. This indicates a distinct and specific role for CDH11 in mediating bacterial adhesion and invasion, rather than a general disruption of the cellular adhesion machinery. This specificity, combined with evidence from Co-IP and computational modeling (docking and MD simulations) predicting a stable direct interaction between CDH11 and FadA, establishes CDH11 as a key host receptor for FadA to facilitate *F. nucleatum* adhesion and invasion into pulmonary epithelial cells. Future studies employing cryogenic electron microscopy, co-crystallization or site-directed mutagenesis will be essential to precisely map the FadA-CDH11 binding interface.

The MAPK signaling pathway plays a central role in pulmonary inflammation and impaired lung function, which are pivotal mechanisms in the pathogenesis of COPD [47,48]. MAPK13 (p38δ), a stress-activated kinase belonging to the p38 MAPK family, is activated by diverse inflammatory stimuli [49]. MAPK activation promotes the transcription and secretion of proinflammatory cytokines and chemokines through phosphorylation of downstream targets such as the AP-1 transcription factor complex, of which JUN is a key component [50,51]. Notably, MAPK13 inhibition has been shown to attenuate airway inflammation [52–54]. This study demonstrates that *F. nucleatum* activates a pro-inflammatory MAPK13/JUN pathway in pulmonary epithelial cells via specific FadA–CDH11 interaction, resulting in upregulated expression of CSF3, TNF-α, CCL20, and TGF-β. Among the upregulated cytokines, TNF-α and TGF-β contribute to a pro-inflammatory and pro-fibrotic positive feedback loop that accelerates lung function decline [55–58], while CSF3 and CCL20 synergistically promote immune cell recruitment, enhance neutrophil and dendritic cell infiltration, and exacerbate airway inflammation [59,60]. These cytokines consistently show an inverse correlate with FEV_1_% pred in COPD [55,56,59,60]. Moreover, the upregulation of CDH11 in COPD lungs was further validated by an independent patient cohort (GSE47460), and increased CDH11 expression was associated with a decline in pulmonary function indices in these patients. Therefore, these findings imply that the FadA–CDH11–MAPK13/JUN axis serves as a key mechanism by which *F. nucleatum* exacerbates pulmonary inflammation and promotes COPD progression. Notably, CDH11 is essential for this inflammatory signaling but dispensable for FadA-mediated p53 suppression, indicating a separate pathway for this oncogenic event. Further studies should focus on elucidating the p53 suppression mechanism and evaluating the therapeutic potential of blocking FadA-CDH11 binding in ameliorating COPD exacerbations driven by *F. nucleatum* infection utilizing mouse or organoid models.

**Fig 7:**
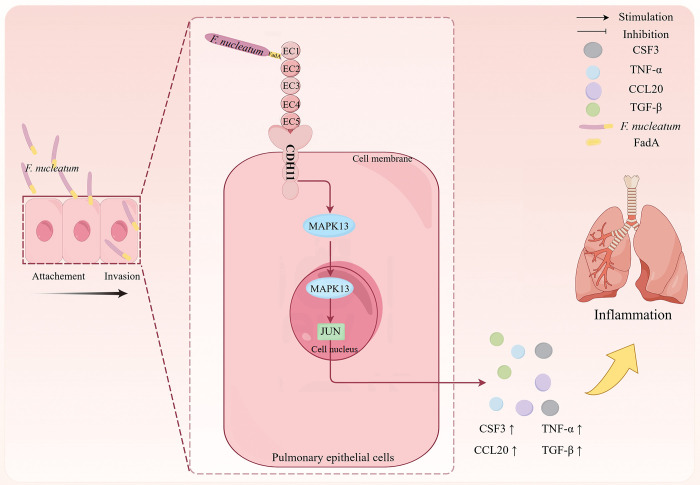
Schematic illustration of the FadA–CDH11–MAPK13/JUN axis mediating *F. nucleatum*-induced pulmonary epithelial inflammation. *F. nucleatum* binds to CDH11 on pulmonary epithelial cells via its adhesin FadA, facilitating bacterial adhesion, invasion and activation of the MAPK13/JUN pathway. This signaling cascade leads to upregulated expression of pro-inflammatory cytokines—including CSF3, TNF-α, CCL20, and TGF-β in pulmonary epithelial cells. Fig 7 is drawn using Figdraw (https://www.figdraw.com).

This study elucidates a critical mechanism by which *F. nucleatum* exacerbates pulmonary inflammation through the FadA–CDH11–MAPK13/JUN axis (Fig 7). Specifically, FadA mediates bacterial adhesion to and invasion of pulmonary epithelial cells through binding with CDH11, a cadherin significantly upregulated in COPD lungs and in pulmonary epithelial cells treated with *F. nucleatum* or FadAc protein. This engagement triggers MAPK13/JUN-driven pro-inflammatory signaling, leading to the expression of cytokines such as CSF3, TNF-α, CCL20, and TGF-β, which collectively enhance immune cell infiltration, accelerate lung function decline, and correlate negatively with FEV_1_% pred. Notably, while CDH11 is essential for inflammatory activation, it is dispensable for FadA-mediated p53 suppression, suggesting distinct pathways for immune modulation and tumorigenic effects. These findings highlight the potential of targeting the FadA–CDH11 interaction as a therapeutic strategy to mitigate *F. nucleatum*-induced pulmonary inflammation and COPD exacerbation.

## Materials and methods

### FadAc protein purification and antibody production

The FadAc protein was obtained using previously described purification methods [17]. Briefly, the gene sequence encoding the FadAc domain of *F. nucleatum* ATCC 25586 was synthesized by Sangon Biotech Company (Shanghai, China), and subcloned into the prokaryotic expression vector pET21(b). Competent *E. coli* BL21(DE3) cells were transformed with the recombinant plasmid pET21(b)-FadA for heterologous expression. *E. coli* cultures grown to OD600 of 0.5 were induced by 0.5 mM IPTG for 2.5 h. The recombinant FadAc protein was purified using a His-Tagged Protein Purification kit (Thermo Scientific, USA) and dialyzed against 10 mM Tris-HCl (pH 7.4) at 4°C. Protein concentration was determined using the bicinchoninic acid Protein Assay Kit (Thermo Scientific, USA).

The polyclonal antibody against *F. nucleatum* or FadA were obtained after immunizing New Zealand white rabbits with inactivated *F. nucleatum* or purified FadAc protein for 7 weeks, respectively [61]. Serum was collected periodically to monitor antibody titers. Polyclonal antibodies were purified from serum using protein A/G affinity chromatography, followed by antigen-specific affinity purification to enhance specificity.

### Antibodies

The primary antibodies used in this study included anti‐GAPDH (1: 800; Proteintech, China), anti‐CDH11 (1: 5000 in western blot; 10 μg/mL in the antibody blockade assay; Abcam, Cambridge, MA, USA), IgG (10 μg/mL; Proteintech), anti‐MAPK13 (1: 500; Proteintech), anti‐JUN (1: 1000; Cell Signaling Technology, Danvers, MA, USA), anti‐p53 (1: 1000; Cell Signaling Technology, Danvers, MA, USA).

### Bacteria culture and treatment

*F. nucleatum* (ATCC 25586) and *P. gingivalis* (ATCC 33277) were obtained from American Type Culture Collection (ATCC). *F. nucleatum* and *P. gingivalis* was cultivated in trypsin soy broth (TSB. Becton, Dickinson and Company, Sparks, MD, USA) supplemented with 5% defibrinated sheep blood, 10 mg/mL hemin and 5 mg/mL menadione in an anaerobic condition (80% N_2_, 10% H_2_, 10% CO_2_) at 37°C.

To elucidate the functional role of FadA adhesin in *F. nucleatum* infection, an antibody blockade assay was performed as previously described [62]. Briefly, *F. nucleatum* were anaerobically pre-incubated with our custom FadA antibody [61] for 1 h at 37 °C. For control conditions, parallel bacterial preparations were incubated with pre-immune serum under identical conditions. Treated bacteria were then used for subsequent experiments.

### Cell culture and treatment

Human pulmonary epithelial cell line A549 was acquired from ATCC and cultured in Dulbecco’s modified Eagle’s medium (DMEM. HyClone Laboratories, Logan, UT, USA) containing 10% fetal bovine serum (FBS. HyClone Laboratories, Logan, UT, USA), 100 U/ml penicillin and 100 μg/mL streptomycin in a humidified 37°C incubator with 5% CO_2_. All cell-based assays described in this study were conducted at 70–80% cell confluence, unless explicitly stated otherwise.

In an *in vitro* bacterial infection model, A549 cells were incubated with *F. nucleatum* at various multiplicities of infection (MOI, bacteria: epithelial cells) in DMEM without antibiotics. In the antibody blockade assay, A549 cells were pretreated with a CDH11‑specific antibody (10 μg/mL) for 2 h prior to infection with *F. nucleatum* (MOI 100). An IgG isotype antibody (10 μg/mL) served as the control [27,63]. In addition, A549 cells were stimulated with purified FadAc protein at concentrations of 0.1, 1, or 10 μg/mL for 24 h at 37°C. Untreated cells served as the control group.

### Epithelial cell attachment and invasion assay

A549 cells were infected with *F. nucleatum* in DMEM without antibiotics in a humidified 37°C incubator with 5% CO_2_ for 3 h and washed with PBS. For attachment assays (to determine total adhesion and invasion levels), cells were lysed with sterile distilled water for 30 min. Dilutions of the lysate were plated and cultured anaerobically on TSB agar supplemented with 5% defibrinated sheep blood, 10mg/mL hemin, and 5mg/mL menadione to determine CFUs for *F. nucleatum*. For invasion assay, extracellular bacteria were killed with 200 μg/mL gentamicin (Sigma, St. Louis, MO, USA) and 300 μg/mL metronidazole (Sigma, St. Louis, MO, USA) for 1 h. Cellular lysates were diluted and cultured to determine CFUs. The number of bacterial attachment or invasion is equal to CFUs divided by the number of cells. To assess the efficacy of the gentamicin-metronidazole combination in eliminating extracellular *F. nucleatum*, supernatants from A549 cell cultures were collected immediately before and 1 h after antibiotic treatment and subjected to CFU counting.

### Fluorescence staining and CLSM analysis

The adhesion and invasion capabilities of *F. nucleatum* and the localization of FadA on A549 cells were assessed by fluorescence staining and visualized using a confocal laser scanning microscope (CLSM, Leica TCS SP5, Leica Microsystems, Mannheim, Germany). A549 cells were infected with *F. nucleatum* in antibiotic-free DMEM for 4 h. After infection, the medium was removed, and the samples were fixed with 4% paraformaldehyde for 10 min. Following PBS washes, the cells were blocked with 1% BSA for 1 h. Subsequently, the samples were incubated with the primary antibody (the polyclonal antibody against *F. nucleatum* or FadA) overnight at 4°C, followed by incubation with an Alexa Fluor 488-conjugated secondary antibody (1:200 dilution; Proteintech Group, Chicago, IL, USA). Actin filaments were stained using Alexa Fluor 594-phalloidin (Invitrogen, Carlsbad, CA, USA) for 20 min. The nuclei were labeled with 4′-6-diamidino-2-phenylindole (DAPI; Beyotime Biotechnology, China). Finally, the samples were sealed with anti-fade mounting medium (p0126, Beyotime, Shanghai, China) and observed under CLSM. The fluorescence intensity was quantified using the ImageJ software. *P. gingivalis*-treated A549 cells were used as the control for verifying the species specificity of *F. nucleatum* antibody.

To differentiate the subcellular localization of *F. nucleatum* or FadA, a sequential immunofluorescence staining protocol was employed. First, samples were incubated with the primary antibody (the polyclonal antibody against *F. nucleatum* or FadA) overnight at 4°C. Extracellular bacteria/proteins were then labeled by incubation with an Alexa Fluor 594-conjugated secondary antibody. Subsequently, cells were permeabilized with 0.1% Triton X‐100, blocked again, and re-incubated with the same primary antibody overnight at 4°C. Finally, total (both intra- and extracellular) bacteria/proteins were labeled using an Alexa Fluor 488‐conjugated secondary antibody. Cell nuclei were counterstained with DAPI.

To investigate the interaction and co-localization between CDH11 and FadA proteins, a double immunofluorescence co-localization assay was performed. Briefly, cells were fixed with 4% paraformaldehyde and blocked with 1% BSA to prevent non-specific binding. The samples were then incubated overnight at 4°C with one primary antibody (a mouse anti-human CDH11 monoclonal antibody). Subsequently, the cells were stained with Alexa Fluor 594-conjugated goat anti-mouse IgG (red fluorescence) to visualize CDH11. Next, the sample was blocked with 1% BSA again. Following overnight incubation with the other primary antibody (a rabbit anti-FadA polyclonal antibody), the cells were stained with Alexa Fluor 488-conjugated goat anti-rabbit IgG (green fluorescence) to visualize FadA. Nuclei were counterstained with DAPI (blue). Images were acquired using CLSM, and the co-localization degree of FadA and CDH11 was quantified by calculating Pearson’s Correlation Coefficient (PCC). The plugin Colocalization Finder was applied to the red and green channels of the images. A line-intensity scan analysis was applied to determine the colocalization of FadA and CDH11 in A549 cells.

### Data acquisition and differential expression analysis

The National Center for Biotechnology Information Gene Expression Omnibus (GEO) database (http://www.ncbi.nlm.nih.gov/geo) is an open-access database. The GSE1650 dataset, which assessed gene expression using the GPL96 [HG-U133A] Affymetrix Human Genome U133A Array in lung tissues from 18 patients with severe emphysema and 12 patients with mild or no emphysema, was utilized as a screening cohort to identify candidate cadherin expression profiles. The GSE47460 dataset, which assessed gene expression using the GPL14550 Agilent-028004 SurePrint G3 Human GE 8x60K Microarray (Probe Name Version) in lung tissues from 220 patients with COPD and 108 healthy controls, serves as an independent validation cohort. Data normalization and identification of differentially expressed genes (DEGs) was performed using the *limma* package in R (version 4.3.2) [64]. DEGs were defined based on a statistically significant adjusted *P*-value (< 0.05) obtained via *t*-*t*est. Associations between continuous variables were evaluated using the nonparametric Spearman’s correlation test.

### Quantitative reverse transcription-polymerase chain reaction (qRT-PCR)

Total RNA was extracted from A549 cells using RNAiso Plus (Takara, China), and reversely transcribed into complementary DNA using Prime Script RT Reagent Kit (Takara, China). QRT-PCR analyses were performed with SYBR Green Master Mix (Takara, China) on an Applied Biosystems 7500 Real‐Time PCR System (Waltham, MA, USA). The amplification program consisted of an initial denaturation at 95°C for 3 min, followed by 40 cycles of 95°C for 10 s, 60°C for 30 s, and a final dissociation step at 95°C for 15 s. All primers are listed in S1 Table. GAPDH was used as an internal control, and the relative expression of genes were calculated using the 2^−ΔΔCT^ method**.**

### Enzyme-linked immunosorbent assay (ELISA)

Cell supernatants from *F. nucleatum*-infected and uninfected A549 cells were collected to detect the levels of CSF3, TNF-α, CCL20, and TGF-β using corresponding ELISA kits (Cloud-Clone Corp., Houston, TX) following the manufacturer’s instructions.

### Western blot

Total protein was extracted from A549 cells using RIPA lysis buffer containing 1 × protease and 1 × phosphatase inhibitors on ice, and protein concentrations were measured using a BCA Protein Assay Kit. Equal amounts of protein were electrophoresed on 12.5% or 10% SDS-PAGE gels, and transferred to nitrocellulose membranes. The membranes were blocked with phosphate-buffered saline containing 0.1% Tween 20 and 5% skimmed milk and incubated with primary antibodies at 4°C overnight. After incubation with secondary antibody, images were acquired by Odyssey CLX (LICOR, Lincoln, NE, USA) and the protein band was analyzed by Image J software (NIH). Quantification results of specific protein bands were relativized to anti-GAPDH expression, through the equation RI (relative intensity) = specific protein intensity/ GAPDH protein intensity.

### Amino acid sequence alignment

The amino acid sequences for CDH11 (UniProt ID: P55287-1), CDH5 (P33151-1) and CDH1 (P12830-1) were obtained from the UniProt database (https://www.uniprot.org). A multiple sequence alignment of these cadherin proteins was performed using CLUSTAL W (https://www.genome.jp/tools-bin/clustalw) [65]. The secondary structure elements were mapped onto the alignment results utilizing ESPript, vesion 3.2 (https://espript.ibcp.fr/ESPript/cgi-bin/ESPript.cgi) [66].

### Co-immunoprecipitation assay (Co-IP)

A549 cells were subjected to immunoprecipitation (IP) assays using an IP/Co-IP Kit (Thermo Fisher Scientific, Waltham, MA, USA) according to the manufacturer’s instructions. Briefly, A549 cells infected with *F. nucleatum* were homogenized in IP Lysis/Wash Buffer supplemented with protease inhibitors using a bead homogenizer. The lysate was centrifuged at 10,000 × *g* for 10 min at 4 °C, and the resulting supernatant was incubated overnight at 4 °C with rotation in the presence of either control IgG or anti-FadA antibodies that had been pre-bound to washed magnetic beads for 1 h at room temperature under continuous mixing. After incubation, the antigen–antibody complexes were then collected with the beads, the supernatant was also collected, and the beads were washed thoroughly before target antigen elution. Immunoprecipitated proteins were subsequently analyzed by western blot. Total cell lysates without immunoprecipitation were used as positive controls (input).

### Molecular docking

The protein structure of CDH11(AlphaFold ID: AF-P55287-F1) was obtained from UniProt (https://www.uniprot.org), and the structure of FadA (PDB ID: 3ETW) was retrieved from the Protein Data Bank (PDB, https://www.rcsb.org). To identify the most plausible binding mode, initial docking poses were generated and evaluated using multiple protein–protein docking web servers, including HDOCK, ZDOCK, and GRAMM. Based on comparative assessment, GRAMM (https://gramm.compbio.ku.edu/) [67] was selected for the final docking simulation as it predicted a higher binding affinity. The resulting FadA–CDH11 complex was visualized and analyzed using PyMOL 2.2.0 software.

### Molecular dynamic (MD) simulations

The small molecule FadA was parameterized with the GAFF force field using AmberTools22, and RESP (restrained electrostatic potential) charges were calculated with Gaussian 16W. The protein CDH11 was assigned the Amber99sb-ILDN force field. Files of FadA and CDH11were merged to construct the simulation system of the complex, and MD simulations were conducted using GROMACS 2022.3 (https://www.gromacs.org/) [68]. The system was solvated in a TIP3P (transferable interatomic potential with three points model) water box and neutralized with Na^+^ ions. After energy minimization using the steepest descent algorithm, the system underwent 100 ps of number of particles, volume, and temperature (NVT) and number of particles, pressure, and temperature (NPT) equilibration. Given the intrinsic flexibility of CDH11’s long, unstructured loops connecting its rigid domains, an extended simulation time of 200 ns was used to ensure the system reached true thermodynamic equilibrium. Trajectory analysis, including the number of protein ligand hydrogen bonds, RMSD, RMSF, SASA, Rg, and MM/GBSA binding free energy, was performed using GROMACS built-in tools.

### Lentiviral vector transfection

The lentivirus vectors for CDH11 knockdown (shCDH11) and overexpression (OE‐CDH11) were constructed by GeneChem (Shanghai, China). A non-targeting shRNA (shCtrl) and an empty vector (OE‐Ctrl) were used as negative controls. The target sequences for shCDH11 were as follows: shCDH11–1, GCAGATTTGTATGGTTCCAAA; shCDH11–2, CCACTTTCCAACCAGCCAATT; shCDH11–3, GCGCCAAGTTAGTGTACAGTA.

A549 cells at a confluence of 20–30% were transduced with lentivirus at a MOI of 10 in the presence of 1 × HitransG P. The infection rate of lentivirus was detected by flow cytometry. After 72 h of transfection, stable transgenic cells were selected using puromycin. Knockdown and overexpression efficiency of CDH11 were validated by qRT-PCR and western blot.

### Statistical analysis

All experiments were performed in triplicate and repeated three times. Data were presented as mean ± standard deviation (SD). Comparisons between two groups were performed using Student’s *t*-test, while comparisons among multiple groups were analyzed by one-way ANOVA followed by multiple comparisons test using GraphPad Prism version 8.0 (GraphPad Software, La Jolla, CA, USA). A *P*-value of less than 0.05 was considered statistically significant.

## Supporting information

S1 Fig Representative images of colony formation assay for *F. nucleatum* adhesion and invasion. (A) Colony formation assay for the adhesion and invasion of *F. nucleatum* at different MOI (10, 50, 100) in pulmonary epithelial cells. A549 cells were infected with *F. nucleatum* at different MOI (10, 50, 100). (B) Colony formation assay of cell supernatant in the *F. nucleatum* invasion assay. A549 cells were infected with *F. nucleatum* at MOI 100. Supernatants from A549 cell cultures were harvested at two time points: immediately before antibiotic addition (before) and after 1 h of treatment (after), respectively. (C) Colony formation assay evaluating the effect of FadA antibody blockade on *F. nucleatum* adhesion and invasion. A549 cells were infected with *F. nucleatum* (MOI 100) that had been pre-incubated with either anti-FadA (1:100) or pre-immune serum (control, 1:100). Fn, *F. nucleatum*.
(DOCX)

S2 Fig Immunofluorescence analysis of *F. nucleatum* and FadA internalization in pulmonary epithelial cells. (A) Expression and localization of FadA in pulmonary epithelial cells infected with *F. nucleatum*. A549 cells were infected with *F. nucleatum* at different MOI (10, 50, 100) or *P. gingivalis* at MOI 100. Green fluorescence indicates FadA labeled with specific antibody, blue fluorescence represents DAPI-stained nuclei, and red fluorescence shows actin cytoskeleton stained with phalloidin. (B) Visualization of *F. nucleatum* internalization via a double-fluorescence assay. Internalized *F. nucleatum* appears green, while extracellular/surface-adherent *F. nucleatum* appears yellow in the merged image. Nuclei are stained with DAPI (blue). (C) Subcellular localization of FadA in pulmonary epithelial cells visualized by dual-immunofluorescence. Intracellular FadA appears green, while extracellular/surface-adherent FadA appears yellow in the merged image. Nuclei are stained with DAPI (blue). Scale bar: 50 μm. ***P < 0.001. Fn, *F. nucleatum*; Pg, *P. gingivalis*.
(DOCX)

S3 Fig Validation of lentivirus-mediated knockdown and overexpression of CDH11 in pulmonary epithelial cells. (A) Efficiency of lentiviral transfection for CDH11 knockdown or overexpression was evaluated using inverted fluorescent microscope and flow cytometer. Scale bar: 100 μm. (B, D) QRT-PCR analysis of CDH11 mRNA expression in A549 cells transduced with lentivirus encoding shRNA targeting CDH11 (shCDH11) or overexpressing CDH11 (OE-CDH11). Scrambled shRNA (shCtrl) and empty vector (OE-Ctrl) were used as negative controls, respectively. (C, E) Western blot analysis of CDH11 protein levels in the corresponding groups. GAPDH was used as a loading control. ***P < 0.001. Data shown in (B-E) are represented as mean ± SD, n = 3 independent samples.
(DOCX)

S4 Fig Representative images of colony formation assay for *F. nucleatum* adhesion and invasion in pulmonary epithelial cells with modulated CDH11 expression or function. (A) A549 cells were pretreated with a CDH11‑specific antibody (10 μg/mL) or an IgG isotype control antibody (10 μg/mL) for 2 h, followed by infection with *F. nucleatum* (MOI 100). Untreated cells served as the positive control. (B) shCDH11 and OE-CDH11 cells were infected with *F. nucleatum* (MOI 100). shCtrl and OE-Ctrl were used as negative controls, respectively.
(DOCX)

S5 Fig CDH11 knockdown or overexpression does not transcriptionally regulate other adhesion molecules. The mRNA expressions of occludin, CDH1, CDH5 and ITGA2 in shCDH11 and OE-CDH11 cells were evaluated by qRT-PCR. A549, shCtrl and OE-Ctrl were used as controls. Data are represented as mean ± SD, n = 3 independent samples.
(DOCX)

S6 Fig Multiple sequence alignment of CDH11, CDH5, and CDH1. Numbers at the top of the alignment indicate sequential amino acid positions. Residue identity and similarity are highlighted as follows: red boxes indicate identical residues; blue boxes indicate residues that are similar and relatively conservative; white characters denote identical residues, red characters represent similar residues, and black characters indicate residues with lower consistency.
(DOCX)

S1 Table Primer sequences for qRT-PCR used in this study. (DOCX)
