# Supplementary material for: Cadherin-11 serves as a novel receptor for Fusobacterium nucleatum adhesin FadA to exacerbate pulmonary inflammation
Source: PLoS Pathog. 2026 Apr 20;22(4):e1014158. doi: 10.1371/journal.ppat.1014158 (PMC13108864; doi:10.1371/journal.ppat.1014158)
Supplement: S1 Fig — (A) Colony formation assay for the adhesion and invasion of F. nucleatum at different MOI (10, 50, 100) in pulmonary epithelial cells. A549 cells were infected with F. nucleatum at different MOI (10, 50, 100). (B) Colony formation assay of cell supernatant in the F. nucleatum invasion assay. A549 cells were infected with F. nucleatum at MOI 100. Supernatants from A549 cell cultures were harvested at two time points: immediately before antibiotic addition (before) and after 1 h of treatment (after), respectively. (C) Colony formation assay evaluating the effect of FadA antibody blockade on F. nucleatum adhesion and invasion. A549 cells were infected with F. nucleatum (MOI 100) that had been pre-incubated with either anti-FadA (1:100) or pre-immune serum (control, 1:100). Fn, F. nucleatum. (DOCX) [file ppat.1014158.s001.docx]

**S1 Fig.**

**

**
