# Supplementary material for: Cadherin-11 serves as a novel receptor for Fusobacterium nucleatum adhesin FadA to exacerbate pulmonary inflammation
Source: PLoS Pathog. 2026 Apr 20;22(4):e1014158. doi: 10.1371/journal.ppat.1014158 (PMC13108864; doi:10.1371/journal.ppat.1014158)
Supplement: S2 Fig — (A) Expression and localization of FadA in pulmonary epithelial cells infected with F. nucleatum. A549 cells were infected with F. nucleatum at different MOI (10, 50, 100) or P. gingivalis at MOI 100. Green fluorescence indicates FadA labeled with specific antibody, blue fluorescence represents DAPI-stained nuclei, and red fluorescence shows actin cytoskeleton stained with phalloidin. (B) Visualization of F. nucleatum internalization via a double-fluorescence assay. Internalized F. nucleatum appears green, while extracellular/surface-adherent F. nucleatum appears yellow in the merged image. Nuclei are stained with DAPI (blue). (C) Subcellular localization of FadA in pulmonary epithelial cells visualized by dual-immunofluorescence. Intracellular FadA appears green, while extracellular/surface-adherent FadA appears yellow in the merged image. Nuclei are stained with DAPI (blue). Scale bar: 50 μm. ***P < 0.001. Fn, F. nucleatum; Pg, P. gingivalis. (DOCX) [file ppat.1014158.s002.docx]

**S2 Fig**.

**

**
