# Supplementary material for: Cadherin-11 serves as a novel receptor for Fusobacterium nucleatum adhesin FadA to exacerbate pulmonary inflammation
Source: PLoS Pathog. 2026 Apr 20;22(4):e1014158. doi: 10.1371/journal.ppat.1014158 (PMC13108864; doi:10.1371/journal.ppat.1014158)
Supplement: S3 Fig — (A) Efficiency of lentiviral transfection for CDH11 knockdown or overexpression was evaluated using inverted fluorescent microscope and flow cytometer. Scale bar: 100 μm. (B, D) QRT-PCR analysis of CDH11 mRNA expression in A549 cells transduced with lentivirus encoding shRNA targeting CDH11 (shCDH11) or overexpressing CDH11 (OE-CDH11). Scrambled shRNA (shCtrl) and empty vector (OE-Ctrl) were used as negative controls, respectively. (C, E) Western blot analysis of CDH11 protein levels in the corresponding groups. GAPDH was used as a loading control. ***P < 0.001. Data shown in (B-E) are represented as mean ± SD, n = 3 independent samples. (DOCX) [file ppat.1014158.s003.docx]

**S3 Fig.**

**

**
