# Supplementary material for: Cadherin-11 serves as a novel receptor for Fusobacterium nucleatum adhesin FadA to exacerbate pulmonary inflammation
Source: PLoS Pathog. 2026 Apr 20;22(4):e1014158. doi: 10.1371/journal.ppat.1014158 (PMC13108864; doi:10.1371/journal.ppat.1014158)
Supplement: S6 Fig — Numbers at the top of the alignment indicate sequential amino acid positions. Residue identity and similarity are highlighted as follows: red boxes indicate identical residues; blue boxes indicate residues that are similar and relatively conservative; white characters denote identical residues, red characters represent similar residues, and black characters indicate residues with lower consistency. (DOCX) [file ppat.1014158.s006.docx]

**S6 Fig.**

**

**
