# Supplementary material for: Cadherin-11 serves as a novel receptor for Fusobacterium nucleatum adhesin FadA to exacerbate pulmonary inflammation
Source: PLoS Pathog. 2026 Apr 20;22(4):e1014158. doi: 10.1371/journal.ppat.1014158 (PMC13108864; doi:10.1371/journal.ppat.1014158)
Supplement: S1 Table — (DOCX) [file ppat.1014158.s007.docx]

**S1 Table.**

| **S1 Table. Primer sequences for qRT-PCR used in this study** | | |
| --- | --- | --- |
| **Gene** | **Forward sequence** | **Reverse sequence** |
| CDH11 | CAATGTGGGAACGTCAGTAATC | CTTCCACCGAAAAATAGGGTTG |
| MAPK13 | CAGCCGTTTGATGATTCCTTAG | GCTGAAGTTCACAATCTCCTTG |
| JUN | CAAACCTCAGCAACTTCAAC | CTGGGACTCCATGTCGATG |
| p53 | TTCCTGAAAACAACGTTCTGTC | AACCATTGTTCAATATCGTCCG |
| CCL20 | ACTTTGACTGCTGTCTTGGATA | ACCCAAGTCTGTTTTGGATTTG |
| CSF3 | CCGACTTTGCCACCACCATCTG | CTGGAAAGCAGAGGCGAAGGC |
| TNF-α | CTCATCTACTCCCAGGTCCTCTTC | CGATGCGGCTGATGGTGTG |
| TGF-β | AGCAACAATTCCTGGCGATACCTC | TCAACCACTGCCGCACAACTC |
| occludin | CTTCCATCCTGTGTTGACTTTG | CACTTTTCTGCCCTGATTCTTC |
| CDH1 | AGAGACTGGGTTATTCCTCC | GGATTTGATCTGAACCAGGT |
| CDH5 | GTACCACCTCACTGCTGTCATTG | CAGGCACGGACGCATTGAAC |
| ITGA2 | GGCTGGCCCAGAGTTTACAT | ATCGCCCCCTCTCCTAACTT |
| GAPDH | CATGTTCGTCATGGGTGTGAAC | GGACTGTGGTCATGAGTCCTTC |
